# The Meat Quality Characteristics of Holstein Calves: The Story of Israeli ‘Dairy Beef’

**DOI:** 10.3390/foods10102308

**Published:** 2021-09-29

**Authors:** Ariel Shabtay, Einav Shor-Shimoni, Ala Orlov, Rotem Agmon, Olena Trofimyuk, Ofir Tal, Miri Cohen-Zinder

**Affiliations:** 1 Beef Cattle Section, Newe-Ya’ar Research Center, Agricultural Research Organization, P.O. Box 1021, Ramat Yishay 30095, Israel; shabtay@volcani.agri.gov.il (A.S.); einav@volcani.agri.gov.il (E.S.-S.); Lab-IASA@excellence.org.il (A.O.); rotema@volcani.agri.gov.il (R.A.); Tropymuk@gmail.com (O.T.); 2 Institute of Plant Sciences, Newe-Ya’ar Research Center, Agricultural Research Organization, P.O. Box 1021, Ramat Yishay 30095, Israel; ofirt@volcani.agri.gov.il

**Keywords:** Holstein, beef, imported animals, local breeds, sustainability, meat quality

## Abstract

Global animal production systems are often criticized for their lack of sustainability and insufficient resilience to ensure food security. The ‘farm-to-fork’ approach aims at orienting food systems towards the creation of a positive environmental impact, nutritious, healthy, safe and sufficient foods, and fairer economic returns for primary producers. Many countries rely on an imported supply of live animals to fulfill their needs for fresh meat. In Israel, ~60% of the sources of fresh beef come from the import of live animals. In order to encourage sustainable beef production in Israel, the proportion of local beef should be raised at the expense of imported animals. However, for this to be achieved, the superior performance of local beef should be justified. The current study was conducted to compare between the meat quality characteristics of local (Israeli Holstein; N = 205) vs. imported (Australian; N = 169) animals. Generally, while the imported calves presented a higher dressing percentage (*p* < 0.0001), the local animals were characterized by tenderer meat (*p* < 0.0001), longer sarcomeres (*p* < 0.0001), higher a* color attributes and pH (*p* < 0.001), superior cooking (*p* = 0.002) and thawing loss (*p* < 0.0001), higher intra-muscular fat (IMF) content, and a higher PUFA proportion (*p* < 0.01 and *p* < 0.0001, respectively) and PUFA:SFA ratio. The findings shown herein may provide sound arguments for stakeholders and policy makers to facilitate sustainable local beef production in Israel.

## 1. Introduction

It has become evident that the sustainability of food systems is critical to their resilience to the recurrence of natural disasters and health crises. This principle is being demonstrated by the current COVID-19 outbreak, which triggered disruptions to import/export activities, in parallel with intensifying calls for shorter supply chains and increased local production [1].

The ‘farm-to-fork’ approach is a comprehensive strategy, seeking to address the challenges of sustainable food systems by orienting food systems towards the creation of a positive environmental impact, nutritious, healthy, safe and sufficient foods, and fairer economic returns, particularly for primary producers. Unique to animal production systems, the ‘farm-to-fork’ initiative aims at avoiding carbon leakage through animal imports, reducing the environmental and climatic impact of animal production, and improving animal health and welfare [2]. Key steps towards the fulfillment of these aspirations may involve raising the portion of local animal production at the expense of importing live animals. However, within the global animal production system, as in the case of beef cattle, countries that do not produce sufficient fresh meat rely, to a great extent, on an imported supply of live animals to accomplish their needs.

In Israel, ~60% of the sources for fresh beef come from the import of live animals, mainly from Portugal, Australia and Eastern Europe, and the rest stems from free-range beef herds and local dairy farms [3]. During the past several years, trends in the Israeli preference for fresh beef consumption have changed; driven by health awareness, environmental consciousness and ethical considerations, locally produced fresh beef has been favored at the expense of imported meat [3]. Still, more than 200,000 live beef animals are imported to Israel annually. To encourage more sustainable beef production in Israel, sound arguments should be presented for stakeholders and policy makers to control the portion of imported animals. Since free-range beef animals constitute only a small fraction of the fresh meat production chain, and due to the limitation of space, which can hardly exceed its current capacity, the vast majority of fresh meat supplies could originate from fattened male calves and culled cows from local dairy farms. However, as their potential to produce competitive, high-quality beef products is still undefined, many of these animals are transferred out of the country.

Holstein is the premier dairy breed in Israel. Although these animals have primarily been selected for milk production, in many parts of the world they constitute a significant portion of the beef production chain [4]. In the United States alone, dairy breeds (most notably Holstein) make up a substantial quantity of the local feedlot cattle, with as many as 3 to 4 million calves annually grown to contribute approximately 15–20% of the nation’s beef supply [5]. In Ireland, of the 1.4 million calves born every year in the local dairy herd, approximately 350,000 are Holstein males, which invariably find their way into the beef sector for rearing and finishing [6]. In comparison with traditional beef breeds, Holstein animals are often criticized for their inferior dressing percentage, as a result of their lower muscle-to-bone ratio [7,8], larger fat deposits (e.g., omental and mesenteric fat) and internal organs (e.g., liver), in order to support their greater lactation requirements [9,10]. In spite of the above, the meat sensory qualities (juiciness, tenderness, flavor, shear force and overall acceptability) of Holstein cows and male calves may be evaluated as indistinguishable from, or even superior to, those of traditional beef breeds [10,11,12,13,14,15,16].

Moreover, the genetic architecture of the Holstein breed might highlight its potential to contribute to meat quality phenotypes. Reports from the Animal Quantitative Trait Locus (QTL) database [17] point out the presence of Holstein QTLs associated with milk production traits in the vicinity of the QTLs for meat quality and carcass traits. For example, a QTL associated with milk protein yield on BTA7 in US Holstein cows [18] neighbors two QTLs for meat fat content [19] and meat tenderness [20]. Other QTLs for somatic cell count on BTA24 of Danish Holstein cow [21] overlaps with several QTLs associated with health, production, reproductive traits, and meat and carcass phenotypes [17].

However, the non-supported, yet widespread belief, in scientific studies, that beef of dairy origin is inferior to beef produced from traditional breeds, alongside the rising need, in Israel, to encourage sustainable beef production systems, serves to justify the current study. Herein, we compare and report the meat quality traits of local Holstein vs. imported Australian (*Bos indicus* X *Bos taurus* crosses) male calves.

Our findings indicate the superior meat quality characteristics of local Holstein beef over that of imported Australian calves and, thus, may lay the foundations for the facilitation of sustainable beef production in Israel.

## 2. Materials & Methods

### 2.1. Collaboration

The part of the study that involved the selection of animals and meat samples was carried out in collaboration with Bakar Tnuva Ltd. (Beit Shean, Israel). Bakar Tnuva is a major beef-producing stakeholder in Israel. It possesses feedlots, abattoirs and meat factories throughout the country, and employs nutritionists, veterinarians, economists, and food technologists, thus controlling the entire fresh beef supply chain of local and imported animals, for the local consumption.

### 2.2. Selection of Animals and Meat Samples

Three groups of animals were the source for the meat samples in the present study: *(i)* Israeli Holstein male calves (N = 205), reared and fattened, from weaning to slaughter, in two farms located in the northern part of Israel: Farm 1 (F1; N = 62) and Farm 2 (F2; N = 143), at the age of ~12 months. *(ii)* Farm 3 (F3; N = 169), Australian male calves, imported to Israel at the age of 8–12 weeks. Their genetic background included a mix of *Bos indicus* (mostly Brahman) and *Bos taurus*. The three farms were located in each other’s vicinity. The three farms used similar rearing protocols and dietary design, provided by Bakar Tnuva. The diet was composed of ground corn (38.97%), gluten feed (8.13%), cotton seeds (pima; 3.75%), DDG (7.71%), Ca—salt (1.22%), Vit mix (0.17%), Whey (30.87%), wheat hay (4.38%), and wheat straw (4.82%). At the age of 12 months, one day prior to slaughter, the animals were transferred to Bakar Tnuva abattoir, and slaughtered on the following morning under similar conditions, as a single group. The carcasses were then trimmed and gradually chilled, initially at 18 °C, for several hours (±8 h), to avoid cold shortening, then hanged overnight, at 1 °C, in the chilling room. The individual records of live body weight (BW) at slaughter and carcass weight were provided in real time.

On the following morning, the carcasses were cut between the 12 and 13th ribs, and boned out from the rib to the lumbar sacral junction. The *longissimus dorsi et lumborum (LL)* muscle (the posterior side of the *longissimus dorsi* muscle) was taken off the left half-carcass of each animal, and the subcutaneous fat and epimysium were removed from the muscle. The muscles were delivered to the laboratory in isothermal containers at refrigerated temperature, within 1.5 h, for phenotyping of the meat quality characteristics, as described below.

### 2.3. Muscle Preparation

In the laboratory, the *LL* muscles were immediately cut into 280–300 g steaks. Two of the steaks were sealed in plastic bags and kept in a dry aging refrigerator (at 0–2 °C) for 48 h, then kept at −20 °C for cooking loss (CKL) and Warner-Bratzler Shear Force (WBSF) analysis. A third steak was used for the determination of the pH and color, followed by analyses of chemical composition, sarcomere length (SL), water holding capacity (WHC), total collagen content and fatty acid (FA) profile.

### 2.4. pH and Color

Meat pH was evaluated at 24 (pH ultimate) and 48 h post-slaughtering (p.s.), using a calibrated pH-meter equipped with a spear-head electrode (Meat pH-meter; Hannah instruments, Model #HI99163; Serial #B0083102). While the pH at 24 h was measured directly on the carcasses, between the 12/13th ribs, in the mid region, the pH at 48 was measured in the aged steaks.

The meat color was measured on the steaks 24 h p.s., following the exposure of their surface to room temperature. A Konica Minolta Chroma Meter CR-410 (KONICA MINOLTA) was used to measure the attributes of lightness (L*), redness (a*) and yellowness (b*). The Chroma meter was operated using illuminant C mode. Prior to the measurements, the device was calibrated, using a white tile standard. Ten replicates were taken from every steak, with special care taken to avoid areas of connective tissue or intramuscular fat.

### 2.5. Chemical Composition

The chemical analyses were performed on 70 g pieces of meat. These included the determination of intra-muscular fat content (IMF%) using the Soxhlet method [22], crude protein (CP) content via the Kjeldahl method [23], moisture, and ash [24].

### 2.6. Water Holding Capacity

The water holding capacity was determined according to the Grau-Hamm method [25], with modifications [26]. Briefly, ~0.3 g of ground meat were weighed and placed on laminated plastic white paper, covered with a Whatman filter (No.1). This “cassette” was set between two Plexiglass plates and subjected to a constant pressure of 1 kg for 10 min. The content of the WHC was measured according to the following equation:

(1) % WHC=[((X∗moisture %)−(X−Y))/X]∗100

where *X* is initial weight of the meat before pressing (g), and *Y* is the final weight of the meat after pressing (g).

### 2.7. Warner Bratzler Shear Force

#### 2.7.1. Sample Preparation

The determination of shear force (SF) was performed according to AMSA 1995 [27] and Wheeler et al. [28]. Briefly, extra fat was removed from the surrounding muscle, and the steaks were frozen at −20 °C, in PA/EVOH/PE plastic bags, following 48 h of ageing. Prior to the analysis, the samples were thawed in the plastic bags, under circulating water, then moved to a 72 °C pre-warmed bath for cooking, until their core temperature reached 70 °C [27]. A temperature probe, HI 9061 (Hanna Food care Digital Thermometer, Bedfordshire, England), placed in the geometric center of a steak, was used to monitor the temperature. Following the cooking process, juices were poured out of the bag (for CKL measurement; see Section 2.8). The meat samples were cooled down and stored overnight at 4 °C.

#### 2.7.2. Coring and SF Measurement

Six cores with a diameter of 1.27 cm (0.5 inch) diameter were cut, on the following morning, from the chilled steaks, in parallel to the longitudinal orientation of the muscle fibers, enabling the shearing action to be perpendicular to the longitudinal orientation of the fibers. The cores were sheared using a V-shaped shear blade with a triangular aperture of 60°, attached to an INSTRON Universal Testing Machine (Model 3343 Instron, UK Ltd. High Wycombe, UK), equipped with a 500 N loading cell, at a crosshead speed of 200 mm/min [27,28]. The Warner Bratzler SF values were calculated based on the average of the 6 cores, using Bluehill software. The peak force required to cut through the fibers was expressed in Newtons (N).

### 2.8. Cooking Loss and Thawing Loss

Cooking loss was expressed as the percentage of weight difference before and after cooking, according to the following equation [29]:

(2) $$\%\;Cooking\;Loss=\left[\frac{X-Y}{X}\right]*100$$

where *X* = weight of raw steak and *Y* = weight of cooked steak.

The thawing loss (TL), the loss of meat fluids due to thawing, was determined as described by Honikel 1998 [30].

### 2.9. Sarcomere Length (SL)

The sarcomere length was determined on thawed meat samples, according to the method used by Cross et al. [31]. The solutions for fiber fixation were prepared according to Koolmees et al. [32]. Briefly, samples without tendons were selected in triplicates from each tissue, and excised in small pieces (2.0 cm × 1.0 cm × 1.0 cm), in a longitudinal orientation of the fibers. An incision was made with a scalpel in the middle of each sample. The pieces were placed in 50 mL tubes fixed with 30 mL of 5% glutaraldehyde solution, for 4 h at 40 °C, followed by overnight fixation at 40 °C, with a 30 mL 0.2 M sucrose solution. Thereafter, flat tweezers were used to gently separate long and thin fibers from the samples. The separated fibers (about 15–20) were placed in a mortar that contained 3–4 mL 0.2 M sucrose solution, and ground with the pestle to a consistent “soup”. The sarcomere length was determined by laser diffraction, using a neon-helium laser (HeNe Laser; λ = 632.8 nm), which was mounted on an optics bench with a specimen-holding device and a screen, as previously described by Cross et al. [31]. The length of at least 10 projected sarcomeres was measured with a ruler for each biological sample. The SL was calculated by the following equation, as provided by Cross et al. [31]:

(3) $$\mu =\frac{0.6328\times D\times \sqrt{{\left(\frac{T}{D}\right)}^{2}+1}}{T}$$

in which *D* was the distance, in mm, from the specimen to the diffraction pattern screen and *T* referred to half of the separation distance (in mm) between the diffraction bands.

### 2.10. Total Collagen Content

The determination of the total collagen content was based on AOAC 990.26 [33], with adaptations from Starkey et al. [34]. Briefly, 20 g of meat were removed and trimmed of external fat and connective tissue. The meat was minced into a paste, and frozen in petri dishes for 3 h, at −20 °C, prior to lyophilization. The lyophilized samples were ground into powder, using a mortar and pestle. Triplicates of freeze dried muscle powder weighing 0.10 g were mixed with three ml of 3.5 M H2SO4 for subsequent hydrolyzation at 105 °C, for 16 h. Hydrolysis was terminated by the addition of 1.5 M NaOH to the hydrolyzed filtrate, prior to the determination of the hydroxyproline content, from a standard curve, as in Starkey et al. [34].

The content of Hydroxyproline (H) in the sample was calculated: H = h × 0. 25/m.

In which the h-hydroxyproline content as read from the calibration curve; 0.25—coefficient, based on the dilution factor and transition between the units; and m —weight of sample portion.

To convert hydroxyproline to total collagen, the following equation was used: total collagen, mg/g = H × 8 (with collagenous connective tissue containing 12.5% hydroxyproline, if nitrogen-to-protein factor is 6.25).

### 2.11. Fatty Acid Profile

The analysis of the FA profile was performed on the lyophilized muscles, as previously described [35]. Lipids were extracted from 1 g sample powder in a hexane: isopropanol solvent mixture, in accordance with Hara and Radin [36]. An aliquot of 40 mg of the lipid fraction was trans-methylated in accordance with Christie (1982) [37], with modifications [38]. Gas chromatography of the fatty acid methyl esters (FAME) was performed with a Hewlett Packard 6890 system, equipped with HP Chemstation software for peak integration. We used a Supelco SP-2560, 100-m fused silica capillary column of 0.25 mm i.d., with ultra-high purity helium carrier gas, at a flow rate of 20 mL/min. The injector and flame-ionization detector (FID) temperatures were 250 °C and 260 °C, respectively. The splitting ratio to the detector was 1:50. The oven temperature schedule was as follows: 140 °C for 5 min, T increase to 175 °C at 4 °C/min, constant 175 °C for 25 min, T increase to 220 °C at 4 °C/min, and constant 220 °C for 20 min. The total run time was 70 min. Standard FAME preparations (Sigma-Aldrich) were injected separately to relate the peaks to the FA. The FAME preparations used were methyl esters of: C10:0, C12:0, C14:0, C14:1, C16:0, C16:1, C18:0, C18:1t9, C18:1t10, C18:1t12, C18:1c9, C18:1c11, C18:1c12, C18:2c9c12, C18:3c6c9c12 (γ-linolenic), C18:3c9c12c15 (α-linolenic), C18:2t10c12 and C18:2c9t11 (conjugated linoleic acid; CLA), and C20:4c5c8c11c14 (arachidonic).

### 2.12. Statistical Analysis

All the variables met our assumptions of normality and were compared among the three farms or two breeds, using a one-way ANOVA, followed by a Bonferroni Multiple Comparison Test (*p* < 0.05). Pearson correlations between the meat quality phenotypes were calculated using the CORR procedure. Letters are used in the figures/tables to indicate pairwise differences identified through this analysis. All the statistical comparisons were conducted using SPSS version 21.0.

## 3. Results & Discussion

Sustainable food systems are designed to provide healthy and nutritious food that is available, accessible, and affordable to everyone for generations to come [39]. At the same time, sustainable systems, as engines of growth, nourish a continuous dialog between social, economic, and environmental components by: *(i)* encouraging local production and distribution infrastructures; *(ii)* protecting farmers and other workers (e.g., paying their salaries), consumers and entrepreneurs (e.g., profits or returns on assets); *(iii)* minimizing their negative effect on the natural environment. [40].

Many of these aspects should indeed be taken into consideration while aiming to promote sustainable beef production in Israel. However, in order to encourage stakeholders and decision-makers to set policies that will encourage positive transformations towards sustainable beef production, identifying the “intrinsic” properties of the food system that will ensure that its essential outcomes are continuously maintained [41] is a prerequisite. A cardinal obstacle ahead of this enterprise is the massive import of live beef animals to Israel [3]. Thus, an initial step to facilitate the above initiative would be through uncovering the advantages of local over imported beef production.

In the current study, we compared between key meat quality phenotypes, in the *LL* muscle, of Israeli Holstein and imported Australian male calves.

### 3.1. Carcass Production

Live bodyweight (BW), carcass weight, and dressing percentage are presented in Table 1. Although live BW did not differ among farms, nor between breeds, carcass weight and dressing percentage were significantly higher in the Australian calves (F3) in comparison with Holstein (F1 and F2; *p* ≤ 0.0001; Table 1). Although a slight significant difference in dressing percentage was revealed between F1 and F2 animals, statistical adjustment to the breed effect highlighted the superior carcass yield of the Australian calves (*p* ≤ 0.0001; Table 1). Indeed, crosses of beef X beef or beef X dairy animals are expected to produce heterogeneous progeny with higher growth rates and dressing percentage compared to dairy-bred cattle [42,43,44]. On the other hand, dairy-selected animals (e.g., Holstein Frisian) are known for their higher proportions of non-carcass parts, as external (head/feet/tail) and internal organs, offal fats and gastrointestinal tract [45], resulting from their engagement in the process of milk production [9,46,47,48].

### 3.2. Technological Parameters of Raw and Cooked Meat

#### 3.2.1. pH and Color

Among others, ultimate pH (pH_u_; measured 24 h post-slaughter) is a major technical attribute that drives consumers’ purchasing decisions about meat. It is influenced by different factors, such as individual cows’ genetic background, their on-farm nutritional regime, and a variety of biochemical events occurring pre-and post-slaughter (e.g., the level of stress prior to slaughter and post-slaughter processing) [49].

Differences in initial pH and the rate of its decline mostly affect sarcomere shrinkage, protein denaturation and myofibrillar lattice spacing [50].

In the current study, the pH_u_ values ranged from 5.74 ± 0.12 (F3) to 5.88 ± 0.28 (F1), and were affected by both farm (*p* < 0.0001) and breed (*p* = 0.0002; Table 2). While the pH_u_ of the Holstein calves from F1 and F2 did not differ, statistical adjustment to breed revealed higher values in the Holstein meat (*p* = 0.0002; Table 2). Similar breed and farm effects were also determined 48 h post-slaughter (*p* < 0.0001; Table 2). These results, typical for meat without DFD or PSE syndromes, were in agreement with the pH values obtained in the *LL* muscle in other studies [51,52,53].

The variation in pH_u_ mostly affected the meat color, an important technological and visual property of meat quality [4]. The light reflected from the surface of the meat is of primary importance, as it affects, to a great extent, consumers’ perceptions and, hence, their purchasing decisions [50].

In the present study, the meat color was determined 24 h post-slaughter (Table 2), and included attributes of brightness (L*), redness (a*) and yellowness (b*). These attributes did not differ between Holstein calves from F1 and F2, but varied significantly when compared to Australian calves from F3 (Table 2). Statistical adjustment to breed revealed differences in meat color characteristics (L*, a* and b*) between the two breeds (*p* ≤ 0.0001; Table 2). More specifically, while the Holstein meat had higher redness and yellowness scores, the Australian meat was brighter (*p* < 0.0001; Table 2). The color attributes reported herein were only in relative agreement with those presented by others [4,53,54,55], presumably due to environmental variations, such as on-farm rearing management and dietary regime, especially towards the end of the growing period. However, the most plausible effect seems technological; while in many studies color attributes are determined 14 days p.s., the data presented in the current study refer to 24 h p.s. Nevertheless, the attributes most appreciable to consumers favored the local Holstein meat, predicting its possible preference over imported Australian meat.

#### 3.2.2. Thawing Loss and Cooking Loss

The flow of exudates from the raw meat of the Australian calves was significantly stronger than the Holstein, as evidenced by the measurement of TL (5.82 ± 2.84% and 3.88 ± 1.05%, respectively; *p* < 0.0001; Table 2). Although the farm effect revealed a difference between the TL scores of the Holstein calves from F1 and F2, it could not obscure the significant distinction among breeds, highlighting the superiority of this characteristic in the meat of the Holstein calves. The loss of exudates following a thermal treatment is evaluated as the CKL. Here, the loss of exudates from the meat of Australian calves was higher compared to the meat of the Holstein animals, when both breed (*p* = 0.0017) and farm (*p* = 0.007) effects were studied (Table 2). The CKL values detected in Holstein *LL* muscle were in agreement with those reported by others [4,15,53,54]. Taken together, following major processes of thawing and cooking that indicated smaller proportions of exudate loss in Holstein samples, both the TL and the CKL parameters demonstrated advantages, from which the industry of local beef may benefit [56].

#### 3.2.3. Water-Holding Capacity

Water-holding capacity is defined as the ability of fresh meat to retain its own water during cutting, heating, grinding and pressing and during transport, storage and cooking [57]. Poor WHC results in high drip and purge loss, which may represent a significant loss of weight from carcasses and cuts and may affect the yield and quality of processed meat [58,59].

While no differences in WHC were found when the breed effect was studied (Table 2), statistical adjustment to farm revealed higher WHC values in F1 compared to those in F2 and F3 calves (*p* = 0.002; Table 2). It is noteworthy that the WHC values of the Holstein calves were lower than those previously reported for that breed and muscle [4,53,54]. Based on the above, and as changes in WHC levels may be caused by differences in the volume of the myofibrils, resulting from variations in the muscle’s inter-filament spacing [60], it is tempting to assume that management conditions on farms, rather than genetic factors, may affect this parameter.

As indicated above, WHC is commonly found in association with pH values, postmortem. Specifically, the power of muscle proteins to bind water becomes weaker when pH declines, due to their ‘movement’ towards their isoelectric point [61]. On the other hand, at higher pH, WHC increases due to an increase in the overall negative charge of proteins, resulting in repulsion of the filaments and more space for the water molecules [61,62].

The above trend was also exemplified in our study, where the pH_u_ rate was positively associated with WHC in the Holstein animals (R^2^ = 0.15; *p* ≤ 0.01, data not shown). Within this association, F1 were characterized by higher WHC (45.01 ± 4.65%) compared to F3 calves (43.11 ± 3.40%), which did not differ from the F2 animals (Table 2).

#### 3.2.4. Chemical Composition

The chemical composition of muscles is relatively constant and includes about 75% water, 19–25% proteins, and 1–2% minerals and glycogen. The lipid fraction of muscle, however, may vary greatly between species, individuals and muscles, as well as cuts from the same animal [63,64].

The lipid fraction of muscle tends to vary highly between species, individuals, muscles, and cuts from the same animal [63,64]. Generally, *Bos taurus* types present higher marbling than *Bos indicus* breeds [65]. Examples include Brahman feedlot-fed steers, with an IMF content of 3.1%, in comparison to *Bos taurus* breeds, such as Angus (6.2%) [66,67] and Hereford (8.3%; fed forage with or without supplementation of high energy diet) [68], at similar ages and in the same muscle. Regardless of the divergence between *taurus* and *indicus*, Asian cattle breeds are known for their high IMF content [64]; Wagyu (Japanese Black cattle) and Hanwoo (Korean) feedlot steers had an IMF content of 34.3% and 13.3%, respectively, in their *longissimus thorasis* muscle [69,70].

These inter-breed differences in IMF were also demonstrated in the current study, in which the *LL* muscles of Holstein calves exhibited higher IMF content than those of their Australian counterparts (*p* = 0.002; Table 3). These findings are not surprising in light of the reported comparative marbling physiology, referring to the greater proportion of the marbling fleck area and number of marbling flecks of Holstein beef, at a similar slaughter age—12 months—to that reported here, even in comparison to typical beef breeds [67]. With respect to the positive effects of IMF on meat organoleptic characteristics, such as flavor, juiciness and tenderness, firmness, and overall acceptability by consumers [71], the findings presented herein rank the local breed in an advantageous position to satisfy consumers’ sensory choices [16].

### 3.3. Characteristics of Meat Tenderness: Shear Force, Sarcomere Length and Total Collagen

Tenderness is the most important sensory attribute by which consumers judge the quality of their meat [16]. It is a variable phenotype, mostly influenced by genetic factors, muscle characteristics at slaughter [72], or post-mortem changes induced by ageing [16]. Indeed, inconsistency in meat tenderness is considered a major obstacle facing the beef production industry [73]. The phenotyping of tenderness is performed via sensorial panel testing or instrumental measurements [74], but most often the two are conducted in parallel, and correlations between them are determined [75].

In our study, tenderness, evaluated instrumentally, was significantly higher in the meat of Holstein calves, as judged by the lower SF values, statistically adjusted to the farm and breed effects (41.5 ± 9.66 N vs. 46.5 ± 9.27 N, respectively; *p* < 0.0001; Table 4). Differences in meat tenderness among *Bos taurus* and *Bos indicus* breeds, with the advantage held by the former, are well documented [76]. This phenomenon mostly stems from genetic variations in the gene encoding the calpastatin proteolytic enzyme, which are found in association with the rate and extent of muscle proteolysis postmortem [76,77,78,79]. Both the calpastatin’s rate of activity and meat tenderness are moderately-to-highly-inheritable, and genetically correlated [78,80].

A comparative analysis of the meat quality characteristics among 15 muscles categorized *LL* as one of the most tender (WBSF < 35 N) muscles in Holstein male calves [4], making it a legitimate target for inter-breed comparisons. Accordingly, and based on tenderness classification, established upon the relationship between instrumental measurements and consumer perception [73], the *LL* muscle of the Holstein calves in the current study, may be ranked as tender, while that of Australian may be ranked as intermediate. Similarly, other studies of Holstein animals observed relatively high tenderness in the same muscle [4,16].

Moreover, a comparison between the meat quality characteristics of Holstein and Salers (dual breed, often used for beef) cull cows, following 14 days of ageing, did not reveal differences in sensory qualities, such as tenderness and juiciness [16]. These findings, which are in agreement with Monso’n, et al. [81], indicate that by applying the ageing process to local Holstein meat, its organoleptic characteristics may be improved beyond their genetic potential.

Another attribute of meat tenderness is SL. It is often used as a post-rigor indicator [82], representing a positive association with meat tenderness and WHC [83]. In our study, SL differed significantly (*p* < 0.0001) between the two breeds (Table 4). When the farm effect was studied, longer sarcomeres were measured in the muscles of Holstein calves from both farms (2.22 ± 0.30 µM and 2.10 ± 0.27 µM, respectively), compared to the SL of Australian calves from F3 (1.98 ± 0.32 µM; *p* < 0.0001).

Total collagen content is a key parameter for the evaluation of meat tenderness [84]. The increase in the stability of cross-linking between collagen molecules, which is determined by growth rate, nutrition and genetics [84], affects the toughness of meat [85]. Studies in cattle have shown a large variation in the content of collagen, which differs between muscles, breeds, and animals of different ages. No differences in the content of total collagen were found in the current study, between farms or breeds. The values ranged between 2.70 ± 0.83–2.88 ± 0.64 mg/g (Table 4), and were comparable to or lower than those in other breeds [86,87], including Holstein, that were exclusively reared on grass pasture [88]. The lower amounts of total collagen reported herein might have resulted from the relatively younger age of the calves (~12 months). Indeed, the proportion of maturity to reducible crosslinks increases with age, resulting in less tender meat in older animals [89]. Nevertheless, Archile-Contreras et al. [90] suggested that variations in collagen turnover might be affected by the position of the muscle in the animal’s body and could, therefore, influence meat tenderness. Accordingly, less positional muscles, such as the *longissimus dorsi*, are characterized by reduced collagen concentrations and cross-links, and therefore, produce tenderer meat compared to locomotive muscles, such as *semimembranosus* (SM) [91].

### 3.4. Fatty Acid Composition

The nutritional, health and sensory qualities of meat are, to a great extent, determined by its FA composition, which is influenced by various factors, such as diet, breed, age and the level of fat content in the muscle [92]. With respect to their nutritional significance, the fact that meat is a major source of dietary SFA, which is implicated in diseases associated with modern life [93], has triggered increased interest in ways of manipulating the FA composition of meat. In particular, efforts have been made to increase the dietary ratio of polyunsaturated fatty acids (PUFA) to SFA to above 0.4, and to decrease the ratio of n-6:n-3 (i.e., alpha-linolenic to linoleic acid) PUFA to less than 4 [93]. In spite of some clear effects of diet on the FA composition of tissues [94], a combination of bovine genetic background and dietary manipulation could favor particular FAs [95].

In the current study, we estimated the effects of farm and breed on the profile of FA in the *LL* muscle of Holstein and Australian calves. The proportions of short chain saturated FAs, including capric (C:10; *p* = 0.0012), lauric (C12:0; *p* < 0.0001), myristic (C14:0; *p* = 0.0002), pentadecanoic (C15:0; *p* < 0.0001), palmitic (C16:0; *p* = 0.0052) and heptadecanoic (C17:0; *p* < 0.0001) acids, were significantly higher in the fat of Australian calves, when the breed effect was studied (Table 5). Surprisingly, in spite of the improved meat tenderness of Holstein calves (Table 4), the proportion of their stearic acid (C18:0) was significantly higher (*p* = 0.0008) (Table 5). Stearic acid is indeed known for its high and positive correlation with the melting point of fat, which in turn reflects the firmness of meat. However, the best prediction of firmness was provided by the ratio of stearic acid to linoleic acid (18:0:18:2) [93], which in the present study was lower for the Holstein calves (3.15 ± 0.86 vs. 3.97 ± 1.07; *p* < 0.0001). The total proportion of mono-unsaturated FAs (MUFAs) did not differ between breeds (Table 5). However, while oleic (C18:1n9c; *p* = 0.0261) and C18:1n10c (*p* = 0.016) FA were higher in the meat of Australian calves, vaccenic (C18:1n11c; <0.0001) and C18:1n12c (*p* < 0.0001) FAs were higher in the meat of Holstein calves (Table 5). Vaccenic acid is formed in the rumen as a result of partial bio-hydrogenation, and is a precursor for tissue-conjugated linoleic acid (CLA), a well-recognized, health-promoting FA [96], whose proportions as observed in this study were not in favor of the Holstein calves, presumably due to breed-specific differences in the activity of tissue stearoyl CoA desaturase (SCD). Unlike CLA, the total proportion of PUFA was significantly higher in the *LL* muscle of the Holstein calves (*p* < 0.0001). Specifically, higher proportions were revealed for linolelaidic (C18:2n6t; *p* < 0.001), linoleic (C18:2n6c; *p* < 0.0001), α-linolenic (C18:3n3; *p* = 0.0015), Eicosatrienoic (C20:3n6; *p* < 0.0015) and arachidonic (C20:4n6; *p* = 0.0047) acids (Table 5).

PUFAs are known to possess anticarcinogenic and hypolipidemic properties [97], and to act as modulators of different transcription factors, providing, at least partially, the metabolic link between dietary PUFA intake, health, and the progression of chronic diseases [98]. However, taking into account the health-promoting capacity of PUFA and the deleterious dietary potential of SFA, nutritionists have suggested that the desired ratio of PUFA to SFA should exceed 0.4, while in some meats, naturally, it is around 0.1 [93]. While in accordance with this notion, the PUFA-to-SFA ratio reported herein again highlights the superiority of local Holstein over imported Australian meat (*p* < 0.0001; Table 5).

## 4. Conclusions

**Figure 1:**
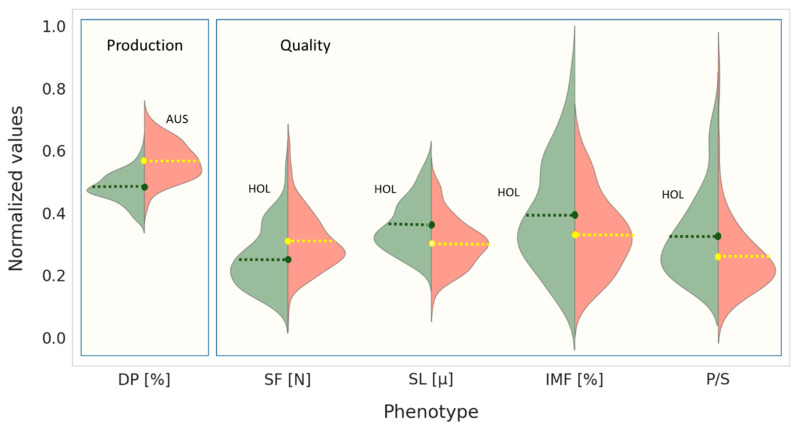
An illustrative violin plot of production (dressing percentage; DP) and meat quality characteristics (shear force, SF; sarcomere length, SL; intra-muscular fat content, IMF; polyunsaturated-to-saturated fatty acid ratio, P/S), comparatively evaluated in Holstein (green; HOL) and Australian (pink; AUS) male calves. The values of each trait are normalized (0,1). Green and yellow dots with dashed lines indicate the mean value of each variable, for Holstein and Australian plots, respectively. The two box plots outlined in blue differentiate between the advantages in production of AUS (left) and meat quality of HOL (quality; right) characteristics.

In summary, from a scientific perspective, the present study provides an understanding of the ‘beefy’ qualities of the dairy Israeli Holstein, via the characterization of the organoleptic, physical and technological properties of its meat. Aiming to lay the foundations for sustainable beef production system in Israel, the current study evaluated the comparative potential of local Israeli Holstein and genetically mixed *Bos indicus* X *Bos taurus* calves, imported from Australia, to produce qualitatively fresh meat. It was found that the meat produced by the local breed was superior according to quality- and health-related characteristics. Specifically, while the Australian calves demonstrated a superior dressing percentage, the Holstein meat was characterized by higher tenderness, greater IMF content, longer sarcomeres, improved PUFA-to-SFA ratio (Figure 1) and superior technological parameters of raw and thermally treated meat.

The results presented herein may provide sound arguments for stakeholders and policy makers to facilitate sustainable local beef production in Israel. Once implemented, such production may comply with the ‘farm-to-fork’ approach by emphasizing potential motives, such as improved animal welfare, traceable, transparent and shorter supply chains, positive environmental impact, the production of nutritious, healthy, safe and sufficient foods, and fairer economic returns for primary producers.

In a broader sense, the current study may serve as an example of how the ‘farm-to-fork’ approach may also be implemented in other parts of the world. In developing countries, for instance, fulfilling the productive potential of imported animals depends upon favorable but unsustainable conditions. Hence, adjusting to an integrative model for sustainable indigenous breed production, which is based on their economic and biological efficiencies, might orient local food systems towards a positive environmental impact, improved product quality and animal health and welfare. Moreover, it may enable smallholder farmers to maintain their animals in the long run, and provide an income for poor farmers while maintaining their cultural identity.

## Figures and Tables

**Table 1:** Carcass production of local Holstein (HOL) and imported Australian (AUS) male calves, adjusted to farm and breed effects. Farm 1 (F1; HOL; N = 62); Farm 2 (F2; HOL; N = 143); Farm 3 (F3; AUS; N = 169).

	*FARM*			*BREED*		*p-Value*	
Trait	F1	F2	F3	HOL	AUS	FARM	BREED
Live BW weight (kg)	519.6 ± 38.0 ^a^	534.8 ± 58.8 ^a^	536.9 ± 91.8 ^a^	530.2 ± 55.5 ^a^	536.9 ± 91.8 ^a^	0.269	0.380
Carcass weight (kg)	279.1 ± 22.6 ^a^	293.0 ± 32.2 ^b^	309.5 ± 58.3 ^c^	288.8 ± 30.3 ^a^	309.5 ± 58.3 ^b^	<0.0001	<0.0001
Dressing percentage (%)	53.7 ± 1.2 ^a^	54.8 ± 2.5 ^b^	57.5 ± 2.1 ^c^	54.5 ± 2.2 ^a^	57.5 ± 2.1 ^b^	<0.0001	<0.0001

Different letters indicate significant differences between farms or breeds (*p* < 0.0001). BW = body weight; Dressing percentage was calculated as the ratio between hot carcass weight and live BW.

**Table 2:** Effects of farm and breed on pH, color attributes, thawing loss (TL), and water holding capacity (WHC) of raw meat, and cook loss (CKL) of thermally treated meat, from Holstein (HOL) and Australian (AUS) male calves. Farm 1 (F1; HOL; N = 62); Farm 2 (F2; HOL; N = 143); Farm 3 (F3; AUS; N = 169).

	*FARM*			*BREED*		*p-Value*	
	F1	F2	F3	HOL	AUS	FARM	BREED
*Raw Beef*							
pH*_u_* ^†^	5.88 ± 0.28 ^a^	5.82 ± 0.21 ^a^	5.74 ± 0.12 ^b^	5.85 ± 0.24 ^a^	5.74 ± 0.12 ^b^	<0.0001	0.0002
pH_48*h*_ ^††^	5.70 ± 0.40 ^a^	5.69 ± 0.20 ^a^	5.53 ± 0.16 ^b^	5.69 ± 0.28 ^a^	5.53 ± 0.16 ^b^	<0.0001	<0.0001
Color							
L*	38.87 ± 3.34 ^a^	38.68 ± 4.14 ^a^	40.97 ± 3.50 ^b^	38.70 ± 3.91 ^a^	40.97 ± 3.50 ^b^	<0.0001	<0.0001
a*	15.85 ± 1.73 ^a^	15.83 ± 1.97 ^a^	14.63 ± 1.93 ^b^	15.83 ± 1.90 ^a^	14.63 ± 1.93 ^b^	<0.0001	<0.0001
b*	3.25 ± 0.98 ^a^	3.02 ± 1.18 ^a^	2.59 ± 1.28 ^b^	3.08 ± 1.10 ^a^	2.59 ± 1.28 ^b^	0.0002	<0.0001
TL (%)	3.30 ± 1.05 ^a^	4.14 ± 2.00 ^b^	5.82 ± 2.84 ^c^	3.88 ± 1.05 ^a^	5.82 ± 2.84 ^b^	<0.0001	<0.0001
WHC (%)	45.01 ± 4.65 ^a^	43.57 ± 3.13 ^b^	43.11 ± 3.40 ^b^	43.68 ± 3.75 ^a^	43.11 ± 3.40 ^a^	0.002	
*Thermally treated beef*							
CKL (%)	22.14 ± 4.40 ^a^	22.12 ± 3.30 ^a^	23.26 ± 3.18 ^b^	22.12 ± 3.66 ^a^	23.26 ± 3.18 ^b^	0.007	0.0017

Different letters indicate significant differences between farms or breeds (*p* < 0.001). ^†^ pH ultimate measured in the carcasses 24 h post-slaughter; ^††^ pH measured in the steaks 48 h post-slaughter; * color attributes measured in the steaks 24 h post-slaughter.

**Table 3:** Effects of farm and breed on proximate composition of meat from Holstein (HOL) and Australian (AUS) male calves. Farm 1 (F1; HOL; N = 62); Farm 2 (F2; HOL; N = 143); Farm 3 (F3; AUS; N = 169).

	*FARM*			*BREED*		*p-Value*	
Trait (%)	F1	F2	F3	HOL	AUS	FARM	BREED
Moisture	73.90 ± 0.76 ^a^	73.59 ± 1.00 ^a^	73.20 ± 1.10 ^b^	73.7± 0.94 ^a^	73.20 ± 1.10 ^b^	<0.0001	<0.0001
Protein	22.92 ± 1.05 ^a^	22.37 ± 0.85 ^b^	22.27 ± 0.85 ^b^	22.47 ± 0.96	22.27 ± 0.85	<0.0001	0.305
IMF	2.78 ± 0.96 ^a,b^	2.82 ± 1.02 ^a^	2.51 ± 0.80 ^b^	2.80 ± 1.00 ^a^	2.51 ± 0.80 ^b^	0.009	0.002
Ash	1.20 ± 0.07 ^a^	1.30 ± 0.18 ^b^	1.27 ± 0.11 ^b^	1.27 ± 0.16	1.27 ± 0.11	<0.0001	0.955

Different lower case letters indicate significant differences between the two breeds for each measurement (*p* < 0.01).

**Table 4:** Effects of farm and breed on tenderness characteristics of the *longissimus lumborum* (*LL*) muscle of Holstein (HOL) and Australian (AUS) male calves. Farm 1 (F1; HOL; N = 62); Farm 2 (F2; HOL; N = 143); Farm 3 (F3; AUS; N = 169).

	*FARM*			*BREED*		*p-Value*	
Trait	F1	F2	F3	HOL	AUS	FARM	BREED
SF (N)	41.3 ± 10.76 ^a^	41.6 ± 9.17 ^a^	46.5 ± 9.27 ^b^	41.5 ± 9.66 ^a^	46.5 ± 9.27 ^b^	<0.0001	<0.0001
SL (µM)	2.22 ± 0.30 ^a^	2.10 ± 0.27 ^b^	1.98 ± 0.32 ^c^	2.14 ± 0.29 ^a^	1.98 ± 0.32 ^b^	<0.0001	<0.0001
Total collagen (mg/g)	2.88 ± 0.64 ^a^	2.81 ± 0.84 ^a^	2.70 ± 0.83 ^a^	2.82 ± 0.82 ^a^	2.70 ± 0.83 ^a^	0.410	0.220

Different lower case letters indicate significant differences between the two breeds for each measurement (*p* < 0.0001).

**Table 5:** Proportion of fatty acids in the *longissimus dorsi et lumborum (LL)* muscle of Holstein (HOL N = 110) and Australian (AUS; N = 100) calves.

Proportion of Fatty Acids	HOL	S.D.	*AUS*	S.D.	*p-Value*
C10:0	0.040	0.0002	0.035	0.0003	1.6 × 10^−1^
C12:0	0.042	0.0003	0.058	0.0004	1.5 × 10^−3^
C14:0	2.716	0.0047	3.242	0.0074	9.2 × 10^−9^
C14:1	0.488	0.0013	0.632	0.0023	1.4 × 10^−7^
C15:0	0.304	0.0006	0.398	0.0012	2.7 × 10^−11^
C16:0	25.56	0.0180	26.27	0.0222	1.2 × 10^−2^
C16:1	3.266	0.0047	3.539	0.0063	4.8 × 10^−4^
C17:0	0.802	0.0028	1.175	0.0024	2.2 × 10^−20^
C17:1	0.428	0.0016	0.723	0.0019	4.9 × 10^−26^
C18:0	17.13	0.0189	16.32	0.0244	7.4 × 10^−3^
C18:1n9t	3.376	0.0193	2.372	0.0093	2.6 × 10^−6^
C18:1n9c	35.02	0.0302	36.12	0.0343	1.5 × 10^−2^
C18:1n10c	1.715	0.0032	1.952	0.0031	1.2 × 10^−7^
C18:1n11c	0.553	0.0019	0.323	0.0016	3.0 × 10^−18^
C18:1n12c	0.317	0.0013	0.387	0.0018	1.6 × 10^−3^
C18:2n6t	0.130	0.0015	0.109	0.0010	2.2 × 10^−1^
C18:2n6c	5.862	0.0174	4.467	0.0172	1.9 × 10^−8^
C20:0	0.041	0.0006	0.000	0.0000	4.5 × 10^−12^
C18:3n3	0.277	0.0008	0.358	0.0015	3.5 × 10^−6^
CLA c9,t11	0.239	0.0015	0.273	0.0011	5.7 × 10^−2^
C22:0	0.237	0.0017	0.000	0.0000	1.7 × 10^−28^
C20:3n6	0.067	0.0016	0.198	0.0015	9.8 × 10^−9^
C20:4n6	1.017	0.0073	0.837	0.0047	3.4 × 10^−2^
C22:2	0.061	0.0013	0.000	0.0000	3.1 × 10^−6^
C24:0	0.000	0.0000	0.086	0.0010	1.0 × 10^−12^
C22:6n3	0.202	0.0026	0.117	0.0022	1.1 × 10^−2^
Short FA	0.082	0.0004	0.093	0.0006	1.2 × 10^−1^
SFA	46.88	0.0217	47.58	0.0415	1.3 × 10^−1^
MUFA	45.16	0.0256	45.75	0.0440	2.5 × 10^−1^
PUFA	7.854	0.0262	6.359	0.0229	1.6 × 10^−5^
PUFA/SFA	0.170	0.0610	0.135	0.0530	2.7 × 10^−5^
C18:0 (stearic): C18:2n6c (linoleic)	3.150	0.8561	3.973	1.0693	7.33 × 10^−9^

FA: Fatty Acid; SFA: saturated fatty acids; MUFA: mono unsaturated fatty acids; PUFA: polyunsaturated fatty acids; S.D: standard deviation.

## Data Availability

The current study does not report any supporting data.
